# Epistatic interactions modulate the evolution of mammalian mitochondrial respiratory complex components

**DOI:** 10.1186/1471-2164-10-266

**Published:** 2009-06-13

**Authors:** Luísa Azevedo, João Carneiro, Barbara van Asch, Ana Moleirinho, Filipe Pereira, António Amorim

**Affiliations:** 1 IPATIMUP-Institute of Molecular Pathology and Immunology of the University of Porto, Porto, Portugal; 2 Faculty of Sciences of the University of Porto, Porto, Portugal

## Abstract

**Background:**

The deleterious effect of a mutation can be reverted by a second-site interacting residue. This is an epistatic compensatory process explaining why mutations that are deleterious in some species are tolerated in phylogenetically related lineages, rendering evident that those mutations are, by all means, only deleterious in the species-specific context. Although an extensive and refined theoretical framework on compensatory evolution does exist, the supporting evidence remains limited, especially for protein models. In this current study, we focused on the molecular mechanism underlying the epistatic compensatory process in mammalian mitochondrial OXPHOS proteins using a combination of in-depth structural and sequence analyses.

**Results:**

Modeled human structures were used in this study to predict the structural impairment and recovery of deleterious mutations alone and combined with an interacting compensatory partner, respectively. In two cases, COI and COIII, intramolecular interactions between spatially linked residues restore the folding pattern impaired by the deleterious mutation. In a third case, intermolecular contact between mitochondrial CYB and nuclear CYT1 encoded components of the cytochrome *bc1 *complex are likely to restore protein binding. Moreover, we observed different modes of compensatory evolution that have resulted in either a quasi-simultaneous occurrence of a mutation and corresponding compensatory partner, or in independent occurrences of mutations in distinct lineages that were always preceded by the compensatory site.

**Conclusion:**

Epistatic interactions between individual replacements involving deleterious mutations seems to follow a parsimonious model of evolution in which genomes hold pre-compensating states that subsequently tolerate deleterious mutations. This phenomenon is likely to have been constraining the variability at coevolving sites and shaping the interaction between the mitochondrial and the nuclear genome.

## Background

The deleterious impact of mutations can be reverted by epistatic interaction with a second-site which acts as a compensatory partner through a process known as compensatory evolution [1]. A detrimental mutation coupled to its compensatory partner results in a compensated background. As long as the deleterious effect of a mutation is neutralized, they find a chance to persist until possibly reaching fixation.

Several examples of compensatory evolution have been reported [2-9], with the great majority of studies involving coevolving nucleotide pairs that participate in the structural folding of RNA molecules. However, theoretical and empirical data are scarce for protein models [10-16]. This remains the case despite the expected impact that a network of intramolecular and intermolecular compensatory epistatic interactions may have on the expression of phenotypes [17] and protein evolution.

Although the most uncomplicated model of compensatory interaction comprises a two-locus, two-allele system, each compensated background may hold more than a single compensatory site for each deleterious mutation [2,18]. In addition, the functional rescue of each particular mutation may involve distinct compensatory solutions [19,20]. A recent study [21], focusing on the structural and physico-chemical properties of compensated mutations, revealed that the probability of a residue being compensated depends upon its location in the protein structure and on the degree of similarity between the changed and the newly arisen residues. That is to say; structurally exposed mutations and replacements involving similar residues are more easily compensated. Overall, these positions seem to represent about 10% of the observed replacements in a protein [12,13,22].

One of the most interesting issues concerning the interaction between coevolving residues is the succession of events that precede the fixation of a compensated genetic background. Two distinct explaining models have been proposed to explain this issue. In one model, a deleterious mutation arises in a background already harboring a fixed or polymorphic compensatory solution [10,12,23]. Because this deleterious effect is suppressed from the moment it occurs, it appears that the occurrence of human deleterious mutations in other mammals [10,12,24] can easily follow this model. Recently, we have shown that a lethal mutation in humans is naturally compensated in chimpanzees. In this case, the ancestral allele acts as a compensatory residue by restoring the protein activity to levels equivalent to the wild-type background [14]. However, unambiguous evidence regarding the recurrence of this model in compensatory evolution still awaits large-scale analyses. In the alternative model, a deleterious mutation occurs first, being subsequently compensated by a second-site substitution. This model presents two possible scenarios. In the first scenario, a mutation persists in the population at low frequencies while waiting for a compensatory solution to arise *de novo *by mutation [25,26], or as a polymorphism that may become associated via recombination [10]. In the second scenario, the fixation of the mutation always precedes the occurrence/fixation of its compensatory partner [27,28]. As expected, this scenario would not apply to strong fitness-affecting mutations.

Herein, the availability of data for a large number of related species motivated the selection of mitochondrial-encoded (OXPHOS) proteins to elucidate the molecular mechanism by which a compensatory residue provides structural, and consequently functional, rescue for the damaging effect of a deleterious replacement through the use of modeled structures. In addition, taking advantage of the large amount of available data for mammalian species, we pursued an extensive phylogenetic analysis to illustrate the sequence of events by which a mutation and a compensatory partner became paired in a compensated background.

## Results and Discussion

### Identification of human deleterious mutations in non-human mammals

At the time of this study, information was available for a total of 199 mammalian species as well as for 49 missense disease-associated mutations occurring at mitochondrial-encoded OXPHOS proteins (detailed information is given in the methods section). We started the analysis by identifying human missense pathological mutations representing the wild-type residue at the homologous site in non-human mammals, following a previously reported methodology [12]. A total of three unambiguously deleterious mutations [29-31], one at cytochrome c oxidase subunit I (COI), another at cytochrome c oxidase subunit III (COIII), and a third at cytochrome b (CYB) were identified and further examined (Table 1).

**Table 1:** Human deleterious mutations at mitochondrial-encoded proteins present as wild-type amino acid in mammalian orthologues.

**Protein**	**Human mutation** **(associated phenotype)**	**Non-human mutation carriers**
COI (Cytochrome c oxidase subunit I)	Leu196Ile (Epilepsy)	Order: Rodentia Family: Gliridae Species: *Myoxus glis *(Fat dormouse)
		
COIII (Cytochrome c oxidase subunit III)	Phe251Leu (Encephalopathy/MELAS)	Order: Primates Family: Cercopithecidae Species: *Chlorocebus aethiops *(African green monkey); *Chlorocebus tantalus *(Tantalus monkey);*Chlorocebus sabaeus *(Green monkey)*; Chlorocebus pygerythrus *(Vervet monkey)*; Semnopithecus entellus *(Hanuman langur)*; Macaca mulatta *(Rhesus monkey)*; Macaca sylvanus *(Barbary ape)
		
CYB (Cytochrome b)	Gly251Ser (Exercise intolerance)	Order: Primates Family: Hominidae Species: *Pongo pygmaeus *(Bornean orangutan);*Pongo abelii *(Sumatran orangutan)
		
		Order: Primates Family: Cercopithecidae Species: *Macaca sylvanus *(Barbary ape);*Colobus guereza *(Guereza);*Trachypithecus obscures *(Dusky leaf monkey);*Rhinopithecus roxellana *(Golden snub-nosed monkey);*Presbytis melalophos* (Mitred leaf monkey);*Procolobus badius *(Western red colobus);*Semnopithecus entellus *(Hanuman langur)
		
		Order: Diprotodontia Family: Phalangeridae Species: *Trichosurus vulpecula *(Silver-gray brushtail possum)
		
		Order: Diprotodontia Family: Phascolarctidae Species: *Phascolarctos cinereus *(Koala)

The COI-Leu196Ile mutation was detected in a patient suffering from epilepsia partialis continua [31]. Comparison of protein sequences revealed that the Leu196 residue is invariant in mammals indicating a critical role for normal protein function. Nevertheless, this mutation overlaps the wild-type residue in a rodent lineage (*M. glis*). The COIII-Phe251Leu mutation, known to be associated with mitochondrial encephalomyopathy, lactic acidosis and stroke-like episodes (MELAS) [29] was observed in seven primate species, all members of the Cercopithecidae family (*C. aethiops, C. tantalus, C. sabaeus, C. pygerythrus*, *S. entellus, M. mulatta *and *M. sylvanus)*. Finally, the CYB-Gly251Ser replacement was detected in a patient presenting paracrystalline inclusions and low aerobic capacity [30]. This deleterious Ser251 residue was found in nine primate species (*P. pygmaeus, P. abelii, M. sylvanus, C. guereza, T. obscures, R. roxellana, P. melalophos, P. badius *and *S. entellus) *as well as in two marsupial species in the Diprotodontia order (*T. vulpecula *and *P. cinereus)*.

### Uncovering mutation-compensation pairs

We next concentrated on the identification of the most likely compensatory site under the previous assumption [12] which stated that if a mutation is deleterious in humans but neutral in related species, protein sequence comparisons should reveal a compensatory site that distinguishes human and non-human wild-type sequences. That is, a compensatory residue must be recognizable in non-human mutation carriers and simultaneously be absent from the normal human sequence.

Here we propose a further extension to these assumptions namely, if a mutation is as deleterious in non-human mammals as it is in humans unless paired with a compensatory partner, the corresponding compensatory residue should be able to rescue the impairment of the human protein. In this perspective, the identification of the most likely compensatory partner for each mutation was initially based on comparative sequence data and subsequently complemented with 2D and 3D structural analyses to allow the recognition of the molecular basis of interactions. These analyses resulted in a vast list of potential compensatory sites for each mutation [see Additional file 1] from which the interacting residues in close spatial vicinity [12,15,16,18,32-34] were selected. Because the crystal structure of the human COI, COIII and CYB proteins has not been solved thus far, we built the structural models using previously established structures of bovine proteins [35,36] as templates (detailed information is given in the methods section).

**Figure 1:**
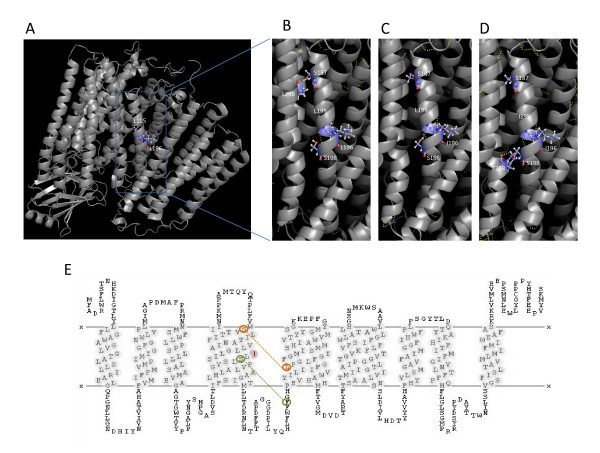
**Molecular mechanism of compensation at COI**. (A) Three-dimensional model of human cytochrome oxidase c complex showing Leu195 and Leu196 positions in COI. (B) Detailed view of the wild-type background (195Leu-196Leu) showing side-chain bonding involving Ser187 (α-helix V) and Leu248 (α-helix VI). (C) Deleterious background (Leu195-Ile196) revealing the absence the H-bonding between α-helices V and VI. (D) Compensated background (Ile195-Ile196) establishing a novel H-bond involving Ser198 (α-helix V) and Phe238 (α-helix VI). (E) Transmembrane structure of the human wild-type COI showing the mutation position (red), the interchain connection in the wild-type background (orange) and the new interaction in the compensated background (green).

Under these models, the compensatory residue for the deleterious COI-Ile196 found in *M. glis *was predicted to be at position 195 (Table 2) represented by an isoleucine in this lineage whereas all the other mammals preserve the leucine. Additional analyses provided compelling support for the compensatory role of Ile195 when interacting with deleterious Ile196. In the human modeled wild-type background (Leu195-Leu196, Figure 1A), side-chain interactions between α-helix V and VI are achieved, at least partially, through an H-bond involving Ser187 (α-helix V) and Leu248 (α-helix VI) (Figure 1B and Figure 1E). This bonding was not predicted in the deleterious background (Leu195-Ile196, Figure 1C), strongly indicating an impairment in protein folding, a hypothesis substantiated by functional evaluations of the affected patient [31]. The connection between α-helices V and VI was restored when the conserved Leu195 was replaced by an Ile, a combination that represent the compensated background (Ile195-Ile196, Figure 1D and Figure 1E). Two interesting points arise here. First, the novel interaction also involves novel intervening residues (Ser198 and Phe238) apart from the mutation (Ile196) and the compensatory residue itself (Ile195). Second, the compensatory residue does not intervene directly to maintain the connection between the two helices, although interaction with other residues seem to contribute to maintain proper protein folding [37]. The reestablishment of the original bonding pattern leading to the rescue of a deleterious phenotype is a remarkable observation in line with previous documented data [18].

**Table 2:** Candidate compensatory residues for three human deleterious mutations.

**Protein**	**Human mutation**	**Compensatory residues**
COI	Leu196Ile	Ile195
COIII	Phe251Leu	Val254
CYB	Gly251Ser	Pro258, Ser263

**Figure 2:**
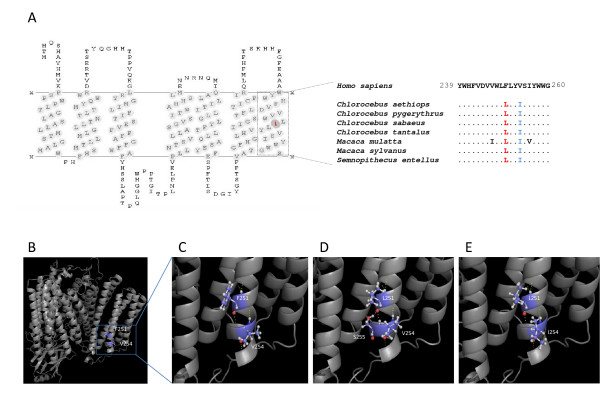
**Molecular mechanism of compensation at COIII**. (A) Transmembrane structure highlighting the deleterious Leu251 at the C-terminus region and sequence comparison between human and primate species harboring the mutation and the most likely candidate to compensatory site. (B) Three-dimensional model of human cytochrome oxidase c complex showing positions Phe251 and Val254 at COIII. (C) H-bonding interactions between Phe251 and Val254 in the wild-type background. (D) Deleterious background (Leu251-Val254) showing a *de novo *interaction with Ser255. (E) Compensated background (Leu251-Ile254) illustrating the reestablishment of the original bonding pattern.

For the identification of a compensatory partner for the COIII-Phe251Leu mutation we followed a similar approach. Comparative sequence analysis revealed 14 candidate compensatory residues for the residue 251 (Table 2), 13 of which are common to all of the species carrying the Leu251 mutation. Of these, position 254 emerged as the most likely compensatory site given the spatial proximity in structural models (Figure 2A to 2C). These structures revealed that the human disease-associated background (Leu251-Val254, Figure 2D) holds an extra H-bond with Ser255 that is not seen in the wild-type background (Phe251-Val254, Figure 2C) and no longer observed in the compensated background (Leu251-Ile254, Figure 2E). Also, despite the amino-acid difference in positions 251 and 254 between wild-type and compensated background, the bonding pattern that involves both residues showed to be surprisingly similar (Figure 2C and Figure 2E).

**Figure 3:**
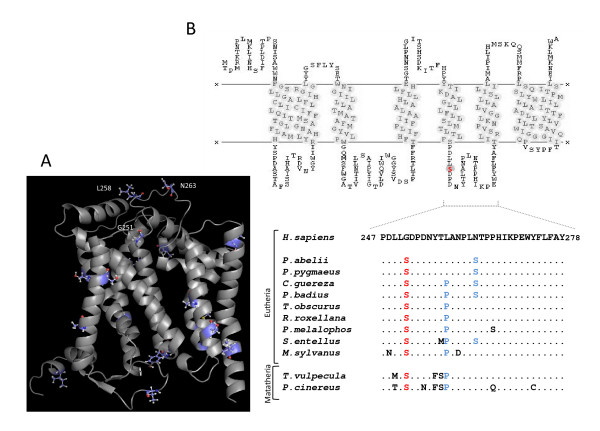
**Molecular mechanism of compensation at CYB**. (A) Human CYB model highlighting the Gly251 residue and all putative compensatory sites [see Additional file 1]. (B) Transmembrane structure with the deleterious residue highlighted and comparison of human wild-type sequence with non-human carriers of the deleterious Ser251 (red) and compensatory Pro258 and Ser263 partners (blue).

Regarding the CYB-Gly251Ser mutation, interspecies sequence comparisons failed to reveal a compensatory residue that was shared by all species carrying the human deleterious Gly251 (Table 2) and this holds true even when placental mammals were considered separately from marsupial lineages. Nevertheless, it is known that more than one compensatory site may exist for any given mutation [19] and thus phylogenetically related lineages can hold distinct compensatory solutions. We mapped onto the modeled 3D structure of CYB all of the possible compensatory alternatives (residues that differ between humans and all of the other mammals) although no structural proximity between each of these residues and Gly251 was evident (Figure 3A).

**Figure 4:**
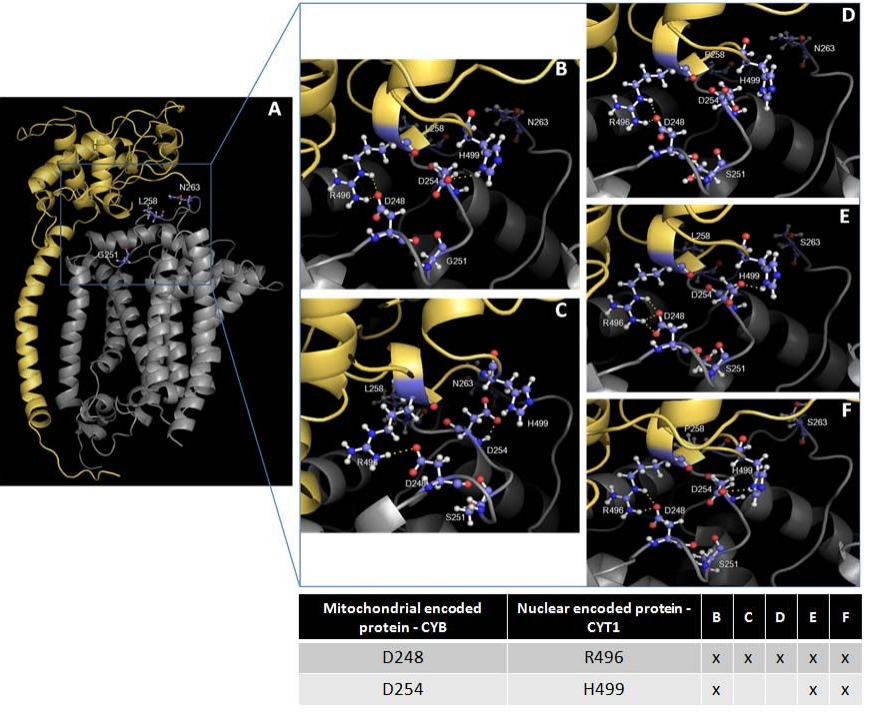
**Mitonuclear interaction between CYB and CYT1**. (A) Human model showing structural proximity between CYB (grey) and CYT1 (yellow). (B) Structure of the wild-type background illustrating H-bond salt bridges across CYB/CYT1 interfaces (CYB-Asp248/CYT1-Arg496 and CYB-Asp254/CYT1-His499). (C) Structure of the deleterious background (CYB-Ser251). (D), (E) and (F) Structure of the compensated backgrounds (CYB-Pro258, CYB-Ser263 and CYB-Pro258-Ser263, respectively). A synopsis of the CYB/CYT1 salt bridge bonding patterns for all of the backgrounds is provided below the structures.

A previous study in yeast [18] revealed that mutations at the mitochondrial CYB can be reverted by distinct compensatory residues and also that each interacting pair lay on the same side of the membrane. To determine whether these evidence apply to the particular case of the Gly251Ser mutation, we focused on the loop that harbors the target residue 251 (Figure 3B). This domain revealed two compensatory candidates, Pro258 and Ser263. Because no direct interaction between mutation and compensatory residue alone or in combination was evident (Figure 3A) and, as argued previously [38], mutations in this strongly conserved domain impairs the complex assembly, we hypothesized that the structural recovery of the mitochondrial *bc1 *complex would require intermolecular interactions with other catalytic subunits, namely the nuclear-encoded cytochrome c1 (CYT1) [39]. In Figure 4, structures representing the mitochondrial-encoded CYB and the interacting region of CYT1 are shown for wild-type (Figure 4A and Figure 4B) and mutated human backgrounds (Figure 4C), as well as for all the possible combinations between the deleterious (251) and the compensatory 258 and 263 residues (Figure 4D to 4F).

Although protein-protein contact is unlikely to be conducted directly by the 258 and 263 residues, the region encompassing CYB/CYT1 interaction would involve H-bond salt bridges between the oppositely charged CYB-Asp248/CYT1-Arg496 and CYB-Asp254/CYT1-His499 residues (Figure 4B). These particular H-bonds are common across protein interfaces [40] and contribute to the stabilization of protein complexes [37,41]. In the mutated background (Figure 4C), one of these bonds (CYB-Asp254/CYC1-His499) seems to be missing, whereas it is recovered in two of the putative compensatory backgrounds harboring Ser263 alone or Ser263 in combination with Pro258 (Figure 4E and 4F). In the remaining background, which harbors Pro258 *solo*, this interaction was not restored (Figure 4D), similarly to what was observed in the deleterious background which may be explained by sequence differences in CYT1 between humans and the more distantly related mammals. These differences may contribute to structural recovery through interaction with the Pro258 residue. Unfortunately, no sequence data related to CYT1 is currently available for these non-human species to confirm this hypothesis. For that reason, this candidate was not included in the subsequent analyses.

To our knowledge, this study provides the first evidence for a compensatory interaction involving proteins encoded by mitochondrial and nuclear genomes in mammals, although a previous study focused on the fitness effect of mitonuclear epistatic interactions in *Drosophila melanogaster *[42] presented evidence that complement our results.

### Coupling of a deleterious mutation with a compensatory partner

After the identification of four mutation-compensation pairs, we attempted to reconstruct the sequence of events that coupled a deleterious mutation with a compensatory partner.

The particular case of COI strongly suggested a quasi-instantaneous occurrence of the Ile196 mutation and compensatory Ile195, since either possible intermediate state (Leu195-Ile196 or Ile195-Leu196) could only have reached fixation at a prohibitively high fitness cost. In one case (Leu195-Ile196) the deleterious impact of the Leu196Ile substitution is well-documented [31]. The other possible intermediate state (Ile195-Leu196) would imply the replacement of an invariant residue (Leu195) in mammals, fishes, amphibians and reptiles, signaling its critical role to the protein function and put on evidence the constraints in replacing leucine for an alternative amino acid. Thus, each of these intermediate states would only be tolerated in heteroplasmy at low frequencies before reached fixation. It is worth noting that replaced residues (195 and 196) lie in the immediate vicinity but do not involve contiguous nucleotides. Thus, it is not obvious whether both nucleotide changes (the first position of each codon) have resulted from the same error during replication or, alternatively, have occurred sequentially towards the rapid fitness escape from unfavorable combinations [43]. But regardless of the mechanism of origin, a quasi-instantaneous fixation of the compensated background (homoplasy) is possible to have occurred within a small number of generations [44,45].

**Figure 5:**
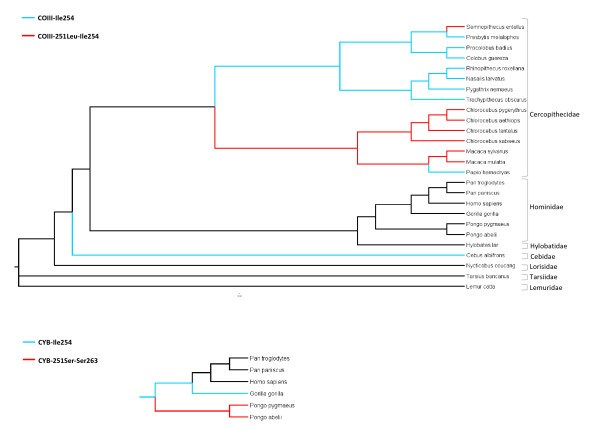
**Maximum likelihood tree showing lineages harboring compensatory residues alone (blue) and compensated backgrounds (red)**.

The deleterious mutations in COIII and CYB were observed in multiple species allowing for the reconstruction of their evolutionary history using a perfectly resolved primate phylogenetic tree (Figure 5). We verified the presence of the candidate compensatory residues in several lineages without the deleterious mutations, but the opposite was never observed. Moreover, it was possible to track at least two independent events resulting in the deleterious COIII-Leu251, but again only in lineages harboring the corresponding compensatory solution. Our previous data also showed that a deleterious mutation in a nuclear-encoded protein (OTC) occurred independently in chimpanzees and dogs. In this case, the compensatory solution is the ancestral amino acid [10]. From these examples, we are presenting a model of co-evolution in which genomes hold potential compensatory solutions for upcoming deleterious events.

**Figure 6:**
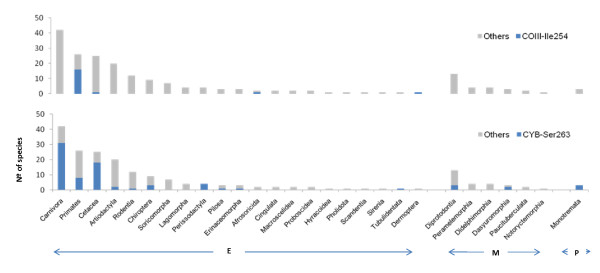
**Distribution of pre-compensating mammalian genomes**. The Eutheria (E), Metatheria (M) and Prototheria (P) orders are represented in the X-axis. The number of species is presented at the Y-axis.

In order to investigate whether other mammalian lineages could also have their genomes pre-compensating for COIII-Leu251 and CYB-Ser251 mutations the phylogenetic analysis was extended. The results, based on the analysis of all mammalian orders included in NCBI dataset, are graphically represented in Figure 6. The widespread occurrence of compensatory COIII-Ile254 and CYB-Ser263 across mammalian orders provides further support that these residues arose through independent mutational events. The remaining CYB-Pro258 residue is invariant in all mammalian orders, except for primates, and represents the ancestral residue as demonstrated by the perfectly resolved deepest branch of the tree (Figure 5B). This is similar to the case mentioned before [10].

## Conclusion

The role played by compensatory processes during protein evolution may now be understood at a greater level. This is aided by the emergence of more sophisticated theoretical and computational prediction tools and increasing empirical evidence. A mutation-compensation pair defines a structural and a functional example of coevolving sites in which the deleterious effect of the mutation is suppressed by its compensatory partner.

In this study, our efforts were directed towards the understanding of the molecular and evolutionary processes of compensation in three highly deleterious mutations that are the wild-type residue in non-human mammalian lineages. In two cases (COI-Leu196Ile and COIII-Phe251Leu), the reestablishment of the original bonding pattern in compensated backgrounds was observed through the extensive analysis of human modeled 3D structures. In the third case (CYB-Gly251Ser), no explicit interactions were observed between the mutation and any of the putative intramolecular compensatory partners. Nevertheless, as both are located in a critical region for the assembly of the *bc1 *complex, we were able to infer the interaction between mitochondrial CYB and nuclear CYT1 in a noteworthy process of intermolecular epistatic interaction between the two genomes.

Although our analyses focused on mitochondrial-encoded proteins, the main conclusions may be extended to nuclear-encoded proteins as well. Under the assumption that a deleterious mutation occurs in pre-compensating nuclear background, one may explain how dominant and fitness-costly mutations are tolerated and reach fixation in various lineages. Furthermore, highly deleterious, yet recessive, mutations may persist in heterozygosity until a compensatory partner arises or becomes linked by recombination. However, because even a small rate of recombination leads to increased fixation time of a compensated background under a strong selection pressure [25,26,46], the pre-compensating model would renders more likely the process of compensatory evolution, in particular when highly deleterious mutations are involved.

## Methods

### Sequences and pathological data

Mitochondrial-encoded protein sequences from over 230 mammalian species were extracted from NCBI Organelle Genome Resources [34]. Whenever data for more than one subspecies was available for each species, only one of those was considered resulting in a total of 199 distinctive species. Sequences were aligned with Clustal W software [47].

Mitochondrial mutations associated with non-LHON clinical phenotypes were obtained from the table "MtDNA Mutations with Reports of Disease-Associations-Coding & Control Region Mutations", available at Mitomap [48]. Only missense substitutions were considered. For all cases, the homologous site at the non-human protein was examined. This resulted in a total of nine cases where the human mutation overlaps the non-human homologous site in mammals (ND1-Met1Thr, ND1-Ala4Thr, ND1-Thr164Ala, ND2-Ala331Ser, ND4-Ile423Val, ND6-Val112Met, COI-Leu196Ile, COIII-Phe251Leu and CYB-Gly251Ser). Since no crystal structure was available for the respiratory complex I, only three cases, COI-L196I, COIII-F251L, and CYB-G251S were analyzed in the present study.

### Transmembrane structure model

Transmembrane models were obtained from the Human Mitochondrial Genome Polymorphism Database [49].

### Protein comparative modeling and structure visualization

We used the human sequence of COI (complex IV) and CYB (*bc1 *complex) as templates to search for the highest E-value pdb using BlastP analysis [50]. The resulting codes of bovine structures (pdb 1occ and 1bgy for complex IV and *bc1*, respectively) were then used in MODELLER [51] in order to build human structural models. The accuracy of the predicted 3D human models [see Additional file 2] was estimated using ProSA-web [52], as previously described [53,54]. All of the structures here analyzed were built using MODELLER and are available at [55]. All residue-residue bonds were calculated using Pymol software [56] and all of the structures were visualized using the same software.

### Phylogenetic inferences

The primate phylogenetic tree was created using maximum likelihood calculations estimated with the PhyML algorithm [57] from an alignment of concatenated COIII and CYB protein sequences. The amino acid matrix used was mtRev and the substitution model was assumed to follow an approximate gamma distribution [58]. For testing the reliability of the PhyML tree topology, the original alignment file was converted to nexus format and served as input for the phylogenetic Bayesian software MrBayes [59,60]. The amino acidic substitution model used was Mtmam following a gamma distribution [58,61,62]. Default settings were used for all of the remaining parameters. The statistical evaluation of posterior distribution for likelihood (LnL) was performed for the two independent MrBayes runs in Tracer v1.4 [63]. The resulting topology of the consensus Bayesian tree was identical to that generated by maximum likelihood analysis [see Additional file 3].

## Abbreviations

mtDNA: mitochondrial DNA; NCBI: National Center for Biotechnology Information; OXPHOS: oxidative phosphorylation; COI: cytochrome c oxidase I; COIII: cytochrome c oxidase III; CYB: cytochrome b; CYT1: cytochrome c1

## Authors' contributions

LA, JC and AA conceived the study and main analyses. LA, JC and AM carried out all the analysis. LA, JC, BA, AM, FP and AA analyzed and interpreted the data. LA, JC, BA and AA wrote the manuscript. All authors read and approved the final manuscript.

## Supplementary Material

Additional file 1 **Candidate compensatory residues for human deleterious mutations**. This table provides all the possible compensatory sites for three human deleterious mutations found in non-human mammals. Click here for file

Additional file 2 **Quality evaluation of the modeled structures of human mitochondrial proteins**. This figure presents the evaluation of model quality for the predicted 3D structures of human COI/COIII (A), CYB (B) and CYT1 (C) both overall (left) and locally (right) as estimated in ProSA-web. Both modeled structures showed z score values (black dots) that lie within the cloud, representing experimentally determined features of native proteins by X-ray and NMR analysis. Energy plots show a smooth fluctuation with overall negative energy of residue stretches (green lines) demonstrating that the predicted 3D structures show minimal deviations from normal energy values. Click here for file

Additional file 3 **Consensus phylogeny of primate lineages**. The figure shows the MrBayes consensus tree illustrating primate topology (A), the marginal density of posterior distribution of likelihood (LnL) for first and second MrBayes runs (B) and Tracer statistical results for tree likelihood, TL (tree length) and alpha in first and second run of MrBayes (C). Click here for file
