# Supplementary material for: Epistatic interactions modulate the evolution of mammalian mitochondrial respiratory complex components
Source: BMC Genomics. 2009 Jun 13;10:266. doi: 10.1186/1471-2164-10-266 (PMC2711975; doi:10.1186/1471-2164-10-266)
Supplement: Additional file 1 — Candidate compensatory residues for human deleterious mutations. This table provides all the possible compensatory sites for three human deleterious mutations found in non-human mammals. [file 1471-2164-10-266-S1.doc]

**Table S1. Candidate compensatory residues for human deleterious mutations.**

| Protein | Human mutation | Compensatory sites |
| --- | --- | --- |
| **COI** | L196I | 3I, 4N, 7F, 28M, 29V, 46A, 50D, 52Q, 102L, 109F, 113S, 116S, 136L, 137A, 139A, 147F, 155S, 156A, 177S, 195I, 220L, 221Y, 247L, 248F, 249T, 250W, 253I, 257R, 258T, 275G, 297L, 330G, 332I, 333V, 336P, 338M, 374L, 376Q, 382F, 383T, 389N, 391V, 394A, 401L, 405S, 406N, 407D, 408A, 409W, 415A, 416V, 419V, 421M, 429D, 452T, 453V, 456M, 467I, 469M, 472V, 481E, 484T, 487L, 490T, 496H, 509T, 511V, 513H |
| **COIII** | F251L | 40T, 62V/I, 73T/A, 88I/V, 111Y/C/H, 143T, 157K, 159T, 171I/T, 178I, 184A, 199I, 224L, 254V |
| **CYB** | G251S | 7T/S/A, 46I, 110L/T/V, 164V/L, 181L, 193T, 212P/S/I, 229I/M, 238A/L/I/VT, 258P, 263S, 303T/A/I/V, 306A/M/V/L, 323F/L, 329I/T/V, 358I, 369S/T/A |
