# Supplementary figures and images for: Epistatic interactions modulate the evolution of mammalian mitochondrial respiratory complex components
Source: BMC Genomics. 2009 Jun 13;10:266. doi: 10.1186/1471-2164-10-266 (PMC2711975; doi:10.1186/1471-2164-10-266)

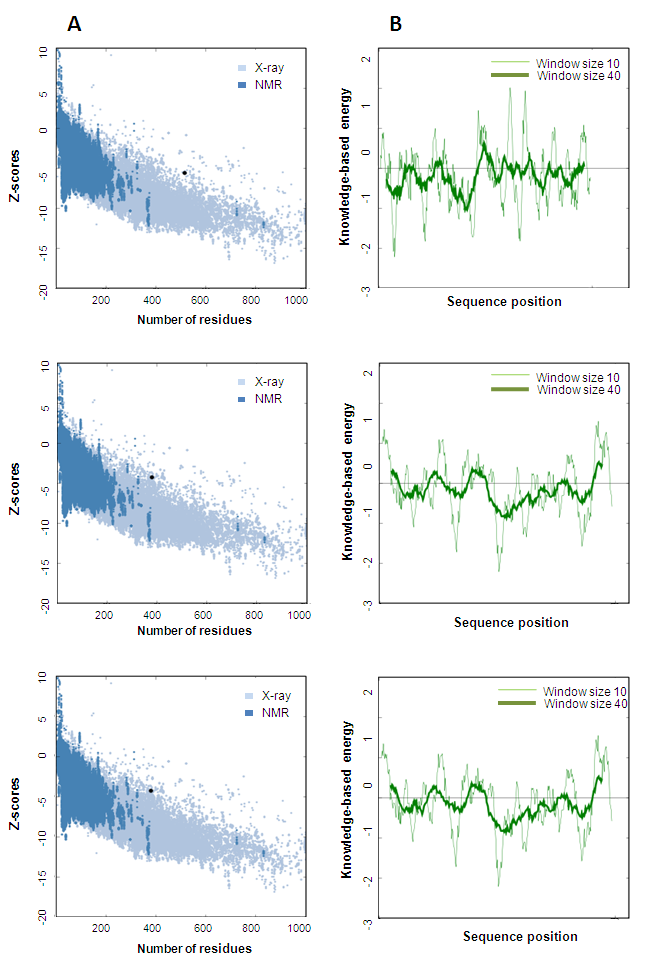

Supplement: Additional file 2 — Quality evaluation of the modeled structures of human mitochondrial proteins. This figure presents the evaluation of model quality for the predicted 3D structures of human COI/COIII (A), CYB (B) and CYT1 (C) both overall (left) and locally (right) as estimated in ProSA-web. Both modeled structures showed z score values (black dots) that lie within the cloud, representing experimentally determined features of native proteins by X-ray and NMR analysis. Energy plots show a smooth fluctuation with overall negative energy of residue stretches (green lines) demonstrating that the predicted 3D structures show minimal deviations from normal energy values. [file 1471-2164-10-266-S2.tiff]

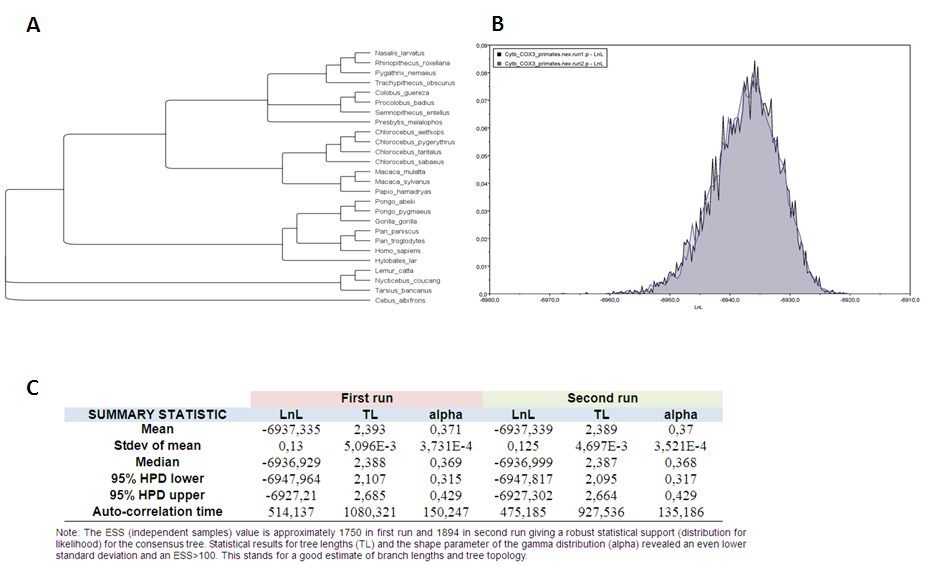

Supplement: Additional file 3 — Consensus phylogeny of primate lineages. The figure shows the MrBayes consensus tree illustrating primate topology (A), the marginal density of posterior distribution of likelihood (LnL) for first and second MrBayes runs (B) and Tracer statistical results for tree likelihood, TL (tree length) and alpha in first and second run of MrBayes (C). [file 1471-2164-10-266-S3.tiff]
